# Traumatische Aniridie: Konservativer oder chirurgischer Therapieansatz?

**DOI:** 10.1007/s00347-021-01367-8

**Published:** 2021-03-29

**Authors:** Christian S. Mayer, Isabella D. Baur, Julia Storr, Ramin Khoramnia

**Affiliations:** 1 Augenklinik, Universitätsklinikum Heidelberg, Im Neuenheimer Feld 400, 69120 Heidelberg, Deutschland; 2 Klinik und Poliklinik für Augenheilkunde, Klinikum rechts der Isar, München, Deutschland

## Anamnese

Ein 14-jähriger junger Patient stellte sich in unserer Sprechstunde aufgrund einer traumatischen Mydriasis und Katarakt vor. Der Patient berichtete, 2 Jahre zuvor durch einen Tannenzapfen eine schwere perforierende Verletzung mit großem Iristeilverlust am linken Auge erlitten zu haben, die extern primärversorgt wurde. Seither sei es zu einer zunehmenden Verschlechterung des Seheindruckes wegen der partiellen Aniridie und einer zunehmenden Linsentrübung gekommen. Zum Zeitpunkt der Erstvorstellung bestand subjektiv ein höherer Leidensdruck v. a. wegen einer ästhetischen Beeinträchtigung und weniger aufgrund vermehrter Blendung. Therapeutisch wurde bereits eine Irisprintkontaktlinse angepasst; er war jedoch mit dem ästhetischen Ergebnis und der Verträglichkeit der Kontaktlinse nicht zufrieden.

## Befund und Diagnose

**Figure 1:**
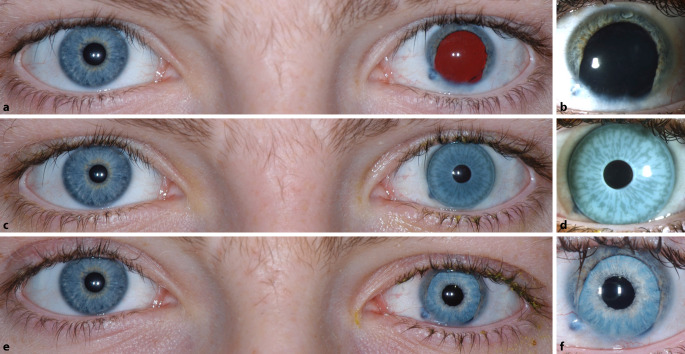

Der bestkorrigierte Visus am betroffenen linken Auge lag bei 0,05 dezimal mit einer subjektiven Refraktion von −3,0/plan/−, am Partnerauge konnten wir einen unkorrigierten Visus von 1,25 dezimal feststellen. In der klinischen Untersuchung zeigten sich am linken Auge neben der traumatischen Mydriasis und dem Irisdefekt bei 5 bis 7 Uhr eine Hornhautnarbe der unteren Hornhauthemisphäre sowie eine traumatische Katarakt. Fundoskopisch zeigten sich links periphere Argonlaser-Koagulationsherde bei ansonsten regelrechtem Befund, jedoch bei reduziertem Einblick aufgrund der Katarakt. Eine Lentodonesis konnte nicht festgestellt werden. Am rechten Auge zeigten sich ein reizfreier Vorderabschnitt mit altersentsprechend klarer Linse sowie ein fundoskopischer Normalbefund. Der intraokulare Druck lag beidseits im Normbereich. Am linken Auge war die Endothelzellzahl mit 1803 Zellen/mm^2^ verringert, wohingegen die Endothelzellzahl am rechten Auge mit 3039 Zellen/mm^2^ normwertig war. Die Kontrastsensitivität wurde mit der Pelli-Robson-Tafel gemessen und ergab für das linke Auge einen Wert von 0,45 „log units“. Der Patient wurde gebeten, die subjektive kosmetische Beeinträchtigung und die subjektive Beeinträchtigung durch Blendung auf einer Skala von 1 bis 10 zu bewerten, wobei 1 für eine geringe Beeinträchtigung und 10 für eine sehr starke Beeinträchtigung steht. Die subjektive Beeinträchtigung durch Blendung wurde vom Patienten mit 2 bewertet, die subjektive kosmetische Beeinträchtigung wurde mit 6 bewertet. Die Abb. 1a, b zeigt den präoperativen Befund ohne und Abb. 1c, d mit Irisprintkontaktlinse.

## Therapie und Verlauf

Der Patient wurde darüber aufgeklärt, dass durch eine Kataraktoperation am linken Auge zwar ein Visusanstieg möglich ist, jedoch die Prognose aufgrund von möglichen (subklinischen) Veränderungen der Netzhaut eingeschränkt ist. Die Möglichkeit einer kombinierten Implantation einer Intraokularlinse (IOL) mit einer Artificial-Iris (AI) wurde mit dem Patienten und den Eltern diskutiert – dies insbesondere im Hinblick auf die Möglichkeit, auf die Iriskontaktlinse zu verzichten, mit derzeitigen Mitteln guten funktionellen *und* ästhetischen Ergebnissen sowie dem gegenüber einer zweizeitigen Operation geringeren Operationsrisiko. Über die möglichen Komplikationen inklusive einer Hornhautdekompensation bis hin zur Notwendigkeit einer Keratoplastik oder der Entwicklung eines Glaukoms wurde der Patient ausführlich aufgeklärt. Bei entsprechendem Leidensdruck entschied sich der Patient für den vorgeschlagenen Eingriff.

**Figure 2:**
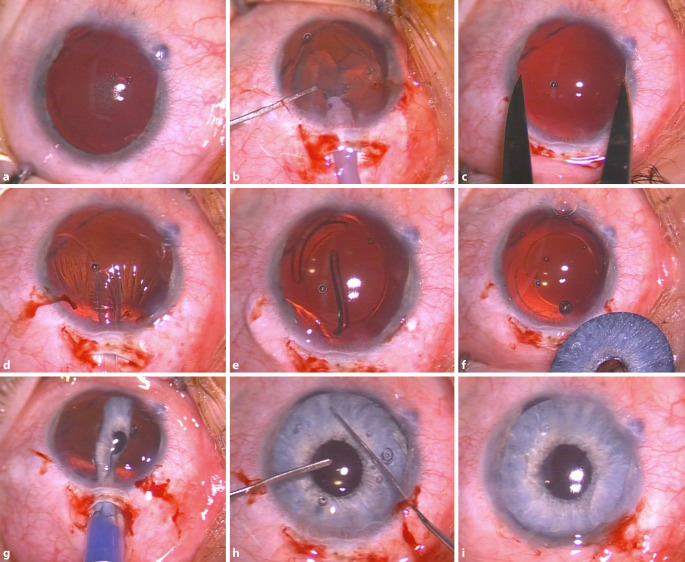

Am linken Auge erfolgte die Implantation einer Intraokularlinse (Aspira-AaY +17,0 dpt., Human Optics, Erlangen, Deutschland), eines Kapselspannringes und der zuvor individuell angefertigten AI (Customflex Artificial*Iris*, Human Optics, Erlangen, Deutschland) in den Kapselsack (In-the-bag-Technik) in einem Eingriff (Abb. 2).

**Figure 3:**
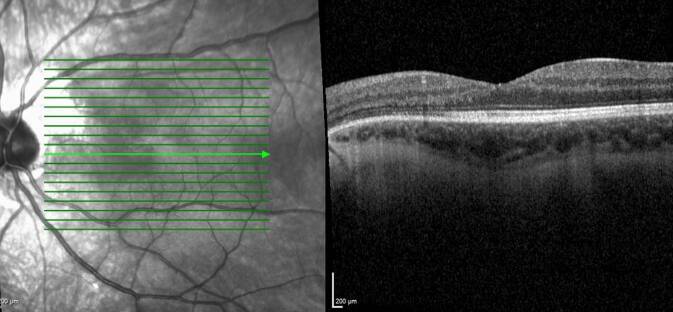

Fünf Monate postoperativ konnten wir am linken Auge einen Anstieg des bestkorrigierten Visus auf 0,32 dezimal mit einer subjektiven Refraktion von +1,5/−1,5/170° feststellen. Die Endothelzellzahl war mit 1753 Zellen/mm^2^ stabil, und der intraokulare Druck lag weiterhin im Normbereich. Die Kontrastsensitivität, gemessen mit der Pelli-Robson-Tafel, stieg auf 1,35 „log units“ an. Postoperativ bewertete der Patient die subjektive Blendung unverändert mit 2, die kosmetische Beeinträchtigung wurde nun mit dem niedrigsten möglichen Wert von 1 bewertet. Postoperativ wurde der Patient gebeten, auch seine Zufriedenheit mit dem Ergebnis auf einer Skala von 1 bis 10 zu bewerten, wobei 1 für geringe und 10 für sehr hohe Zufriedenheit steht. Der Patient gab hierfür den Wert 9 an. Die Abb. 1e, f zeigt das postoperative Ergebnis. Abb. 3 zeigt eine postoperative Spectral-Domain Optische Kohärenztomographie (SD-OCT) des linken Auges.

## Diskussion

Die Versorgung unseres jungen Patienten mit einer AI in Kombination mit einer Intraokularlinse verlief komplikationslos. Wir konnten ein sehr gutes ästhetisches Ergebnis und eine sehr hohe Patientenzufriedenheit beobachten. Auch einen Visusanstieg und eine Verbesserung des Kontrastsehens konnten wir verzeichnen. Obwohl das primäre Ziel bei der AI Implantation nicht die Visusverbesserung, sondern die Reduktion der Blendempfindlichkeit ist, wurde ein Visusanstieg schon mehrfach berichtet, was am ehesten auf die oftmals im gleichen Eingriff durchgeführte IOL-Implantation zurückzuführen ist [1–4]. Es wurde gezeigt, dass durch die AI-Implantation eine signifikante Reduktion des Pupillendurchmessers sowie ein Anstieg des Kontrastsehens erreicht werden können [1, 5].

Die Customflex Artificial*Iris *wird für jeden Patienten individuell angefertigt. Dazu wird die Vorderseite des flexiblen Silikonimplantates von Hand bemalt, um es dem Aussehen des verbliebenen Irisgewebes und des Partnerauges möglichst genau anzupassen. Hierzu ist eine farbgetreue Fotodokumentation vor der Anfertigung zwingend erforderlich. Durch die individuelle Anpassung des Implantates können ausgesprochen gute kosmetische Ergebnisse erreicht werden [2, 5–7]. Gerade junge Patienten legen oftmals besonderen Wert auf das äußere Erscheinungsbild und profitieren bezüglich der subjektiven Beeinträchtigung durch die ästhetische Entstellung von einem rekonstruktiven Eingriff.

Die künstliche Iris kann mit verschiedenen Verfahren in das Auge implantiert werden [8–10]. Bei unserem Patienten konnte die AI zusammen mit einer IOL und einem Kapselspannring in den Kapselsack implantiert werden. Der Kapselspannring soll einer Verkippung der AI durch eine Fibrose und Kontraktion des Kapselsackes vorbeugen. Die Berechnung der IOL-Stärke kann mittels Biometrie ohne Korrekturfaktor erfolgen [11]. Die effektive Linsenposition wird durch die zusätzliche Implantation der künstlichen Iris nur minimal verändert, was die postoperative Refraktion bestätigte.

In manchen Anwendungsfällen wurde ein Anstieg des intraokularen Drucks nach AI-Implantation beschrieben [3, 12]. Patienten mit einem vorbestehenden Glaukom gelten als Risikogruppe für einen postoperativen Druckanstieg, wobei nicht in allen Fällen eine eindeutige Ursache wie ein Pupillarblock oder Pigmentdispersionsglaukom identifiziert werden konnte. Bislang ist noch unklar, ob Iridektomien an der künstlichen Iris einen Pupillarblock wirksam verhindern können [3, 8]. Auch eine okuläre Hypotension kann nach der AI-Implantation auftreten, wobei auch hier Patienten mit einem bereits präoperativ verminderten intraokularen Druck aufgrund eines schweren okulären Traumas häufiger betroffen sind [12]. Ähnlich wie bei einer Kataraktoperation kann die Implantation einer AI zum Endothelzellverlust führen [3, 12, 13], jedoch kann dies bei einer bereits durch ein Trauma reduzierten Endothelzellzahl zu einer Hornhautdekompensation bis hin zur Notwendigkeit einer Keratoplastik führen. Auch eine (Sub‑)Luxation einer sulcus- oder nahtfixierten AI mit dem Risiko einer Luxation in den Glaskörperraum kann auftreten [3]. In diesen Fällen ist eine Reposition, ggf. mit Nahtfixierung der AI erforderlich, um Schäden an der Netzhaut zu verhindern [3, 12]. Neben chronischer Inflammation und zystoidem Makulaödem gehört auch das sog. „residual iris retraction syndrome“ (RITS) zu den bekannten Komplikationen nach AI-Implantation. Beim RITS kommt es aus unbekannter Ursache zu einer progredienten Vergrößerung der natürlichen Pupillenöffnung, die mit einer chronischen Entzündungsreaktion und erhöhtem intraokularem Druck in Verbindung gebracht wird [14]. Das Risiko für Komplikationen hängt maßgeblich vom Ausmaß der Verletzungen eines Auges und der gewählten Technik für die Implantation ab. In unserem Fall mit der Möglichkeit der Implantation in den Kapselsack bei einem verhältnismäßig stabilen präoperativen Befund durften wir von einem eher geringen intra- und postoperativen Komplikationsrisiko ausgehen.

Alternative Implantate sind das Aniridieimplantat Type 68 von Morcher (Stuttgart, Deutschland), das nur in schwarzer Farbe erhältlich ist, oder das Irisimplantat Modell C1/F1 der Firma Ophtec (Groningen, Niederlande), das in ca. 120 verschiedenen Designs hergestellt wird. Beide Implantate sind mit eingearbeiteter Optik erhältlich, haben jedoch den Nachteil, dass das ästhetische Ergebnis aufgrund der eingeschränkten Farbauswahl und fehlenden Individualisierung nicht mit dem in diesem Fall verwendeten Implantat vergleichbar ist. Außerdem ist es nicht möglich, diese in den Kapselsack zu implantieren.

Zusammenfassend stellt die Implantation einer AI zusammen mit einer Intraokularlinse und einem Kapselspannring in den Kapselsack eine elegante Methode dar, um bei einer traumatischen Aniridie und Katarakt Funktion und Ästhetik auch bei sehr jungen Patienten in einer einzigen Operation wiederherzustellen. Gerade bei jungen Patienten muss auch das langfristige Komplikationsrisiko berücksichtigt werden, da das Implantat noch viele Jahre im Auge verbleiben wird.

## Fazit

1. Auf den ersten Blick stellt die Irisprintkontaktlinse eine gute Therapieoption bei einer traumatischen Aniridie dar. Jedoch ist die Anwendung komplex und teuer.
2. Selbst bei vermeintlich guter Optik bei der Aufsicht (Abb. 1c, d) können Patienten über Unzufriedenheit mit Irisprintkontaktlinsen klagen. Die Farbe befindet sich auf der gekrümmten Oberfläche der Kontaktlinse. Dies wirkt insbesondere beim Blick von der Seite auf das Auge des Patienten unnatürlich.
3. Im Falle einer zusätzlich bestehenden traumatischen Katarakt ist eine alleinige Versorgung mit Irisprintkontaktlinsen nicht mehr zielführend.
4. Im Rahmen einer ohnehin durchzuführenden Kataraktoperation kann mit vergleichsweise geringem Aufwand auch die Aniridie behandelt werden. Die Operation ist insbesondere dann risikoarm, wenn die künstliche Iris und die IOL gemeinsam in den Kapselsack implantiert werden können.
